# Effectiveness of Nature Reserve System for Conserving Tropical Forests: A Statistical Evaluation of Hainan Island, China

**DOI:** 10.1371/journal.pone.0057561

**Published:** 2013-02-28

**Authors:** Wei Wang, Peter Pechacek, Mingxia Zhang, Nengwen Xiao, Jianguo Zhu, Junsheng Li

**Affiliations:** 1 Chinese Research Academy of Environmental Sciences, Beijing, China; 2 State Key Laboratory of Environmental Criteria and Risk Assessment, Chinese Research Academy of Environmental Sciences, Beijing, China; 3 The Center for Integrative Conservation, Xishuangbanna Tropical Botanical Garden, The Chinese Academy of Sciences, Menglun, Xishuangbanna, Yunnan, China; 4 Ecology, Conservation and Environment Center, Kunming Institute of Zoology, The Chinese Academy of Sciences, Kunming, Yunnan, China; University of Western Ontario, Canada

## Abstract

Evaluating the effectiveness of existing nature reserve systems for the conservation of tropical forests is an urgent task to save the remaining biodiversity. Here, we tested the effectiveness of the reserve system on Hainan Island by conducting a three-way comparison of changes in forest area in locations within the reserves, adjacent to the reserves, and far outside of the reserves. We used a general linear model to control for the effects of covariates (historical forest area, elevation, slope, and distance to nearest roads), which may also be correlated with the changes in forest area, to better explain the effectiveness of the reserve system. From 2000 to 2010, the forest area inside Hainan’s nature reserve system showed an increase while adjacent unprotected areas and the wider, unprotected landscape both experienced deforestation. However, the simple inside-outside comparisons may overestimate the protective effect of the reserve system. Most nature reserves (>60%) showed increasing fragmentation. And the risk of rapid deforestation remained high at low elevations, where remaining forests tend to be easily logged and converted to commercial plantations. Future conservation efforts should pay more attention to those sites with less challenging environmental conditions.

## Introduction

One of the most common conservation strategies in the protection of tropical forests and mitigation of climate change is the establishment of protected areas (PAs) [1]–[3]. To date, 23% of tropical moist forest and 11% of tropical dry forest around the world are protected [4]. Although the total area set aside for protection continues to increase, it is unclear whether the strategy effectively achieves the stated conservation objectives [5], [6]. In the interest of facilitating the conservation of biodiversity, the Convention on Biological Diversity (CBD) decided to evaluate and improve the effectiveness of PAs in 2004 [7]. Considering that much of tropical biodiversity is unlikely to persist in the face of the growing pressure of human activities, assessing the effectiveness of the PA systems in the conservation of tropical forests is one of the most urgent issues in the preservation of remaining tropical biodiversity [4], [8].

Past studies of the effectiveness of the PAs system have focused on improving representativeness by working on system design and identifying features that were inadequately covered relative to specified targets [9]. However, these studies did not reveal the impact of habitat loss and could have been misleading in terms of historical context [10], [11]. For instance, if a particular habitat takes up 10% of an existing PA system, but 70% of that habitat’s original cover had already been lost at the time of observation, it would be more accurate to say that only 3% of its previous distribution was protected. Merely assessing the representativeness of the PAs system is not enough to determine whether it provides effective protection for tropical forests.

Recently, more studies have focused on how well biodiversity features are actually protected or conserved [12]. One approach is to predict the deforestation that would have been observed had PAs not been established [13], [14]. Empirical studies of this kind are far scarcer than those for representation, and typically rather more limited in scope, largely due to the difficulty of acquiring baseline data [5], [6], [12]. A commonly adopted method is to compare rates of land-cover clearing inside and outside PAs [1], [15]–[18]. One can conclude that the PAs are partially effective at conserving biodiversity when deforestation rates are lower inside than outside PAs. However, this approach may provide somewhat optimistic evaluations of PAs’ effectiveness. This is because the creation of a PA might displace deforestation activities into neighboring forests through preemptive clearing, relocation of displaced communities, and immigration and development along the PAs’ boundaries (“neighborhood leakage”) [5], [19]. In addition, PAs are often located in relatively inaccessible remote areas, which are mostly at higher elevations, with steep slopes, and far away from main roads and residential sites [20]–[23]. For these reasons, the effectiveness of the PA systems should be tested taking environmental and human impact conditions into account [24].

China has been making great efforts toward protecting its natural resources since the first Nature Reserves (NRs) were established in 1956 [25], [26]. NRs are the main body of China’s PA system, but little is known about their effectiveness due to a lack of systematic planning and spatial data on their extent and boundaries [26], [27]. Here we selected Hainan Island, which harbors the most extensive primary tropical rainforest in China [28], to assess the effectiveness of the NR system in the conservation of natural forests. To do so, we (1) compared changes in forest area and fragmentation patterns among the forest patches inside NRs, in adjacent 10-km unprotected areas, and in the wider unprotected landscape from 2000 to 2010; (2) identified the effects of covariates (historical forest area, elevation, slope, and distance to nearest roads) on observed changes in forest area; and (3) determined the effectiveness of the NR system by comparing deforestation rates in protected and unprotected areas while controlling or not for the effects of those covariates. Our results provide information useful for future conservation efforts to maintain tropical forests in Hainan.

## Materials and Methods

### Study Area

**Figure 1:**
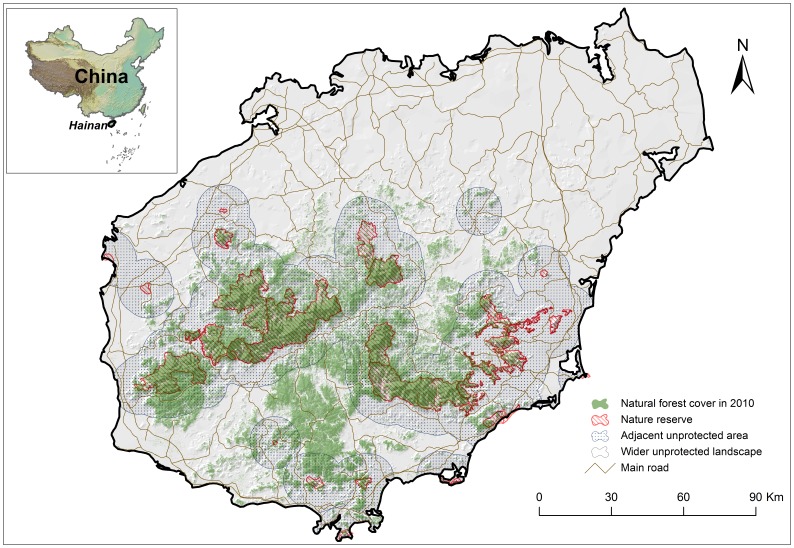
Nature reserve system of Hainan Island. The nature reserve system, adjacent unprotected areas (surrounding lands within 10 km of the nature reserve boundaries) and wider unprotected landscape (more than 10 km away from the nature reserve boundaries) overlaid with natural forest cover in 2010 and digital elevation model (DEM) of Hainan Island, China.

The study was carried out on Hainan Island (Fig. 1), which has an area of about 34,000 km^2^. Hainan Island is located at the northern edge of the Indo-Malayan rain forest (18°09′–20°11′ N, 108°36′–111°04′ E). The island is mountainous in the middle, and flatter in northern and coastal areas. Vegetation is diverse across the island, with a pattern of vertical zonation. In mountainous areas with high rainfall, lowland rainforest occurs below 600 m, montane and ravine rainforest occur between 600 and 1200 m, and evergreen broadleaf forest occurs above 1200 m. Small areas of dwarf mossy forest are distributed on ridges of mountain tops [29].

Since 1949, human activities have caused serious deforestation and degradation of Hainan’s forests [30]. To protect the intrinsic biodiversity of Hainan, the clear-cutting of all rainforests was banned in 1994 [31]. Although human population increased rapidly during the past 20 years, the reforestation of degraded land and the reduction in logging of natural forest have had a positive effect on maintaining forests [31]. Currently, 8.4% of Hainan is set aside in 54 NRs [32], mainly located in remote and economically less valuable areas [33], which is similar to most NRs of China [26]. Human disturbance within the NRs was limited except for occasional hunting and gathering by the indigenous people in the island [28]. However, it is unclear whether the current NR system effectively protects Hainan’s forest cover or if the protection is merely due to challenging topography consequently associated with less human pressure. This uncertainty makes it difficult to develop flexible management and funding mechanisms for future conservation actions.

In this study, we excluded 20 marine and wetland NRs and six NRs with an area <100 ha, focusing on the remaining 28 NRs that are primarily dedicated to protecting forest ecosystems (Table S1). These NRs had been established for at least five years, which is long enough for them to reflect recent management activities [6]. In fact, most of them were established between 1974 and 1996, only three were established in 2004 and two were established in early 2006. We delineated boundaries of the NRs as polygons using the NRs’ management plans (from the Department of Land Environment & Resources of Hainan Province), supplemented by measurements taken in the field.

### Forests Mapping

We used eight Landsat TM/ETM+ images, four from 2000 and four from 2010, covering all of Hainan Island (path/row numbers 123–124/46–47) to obtain information on land cover. We downloaded these images from the International Scientific Data Service Platform, Computer Network Information Center of the Chinese Academy of Sciences website (http://datamirror.csdb.cn). All images had a resolution of 30 m and were geo-referenced to Gauss Kruger/Krasovsky coordinates with a root mean square <1 pixel.

In order to collect ground data for both mapping and validation, we employed a stratified method to identify 1225 samples of 100×100 m^2^ across the whole island. A full description of the sampling process can be found in Zhang et al. [29]. We conducted two field surveys in 2005 and measured the canopy cover and tree height in each sample, using handheld Garmin 72 GPS receivers to record the location for the ground truth data. Using vegetation class definitions issued by the International Geosphere–Biosphere Programme (IGBP), we defined forests as those areas dominated by natural trees with a canopy cover >60% and mean height exceeding 2 m, covering at least 1 ha [34]. Plantations with simple grid-like and homogeneous structures were considered distinct from these “natural” forests and as one type of non-forest land cover. In the field surveys, we collected only the data that showed minimal change from 2000 to 2005 and ignored other data, using information from local forestry bureaus and nature reserves administrations. We used these ground truth data to develop and assess the 2000 forest map. To develop and assess the 2010 forest map, we compared ground truth data collected in 2005 to high-resolution Google Earth images from 2010 and treated unchanged areas as ground truth data. We randomly selected about half of the ground truth data and kept them as training data for classification. We used the remaining data for assessing the accuracy of the forest maps.

We used a maximum likelihood classification algorithm to classify 2000 and 2010 images separately using Erdas Imagine 9.0 (Leica Geosystems Geospatial Imaging LLC, 2005) with the aid of training data and Digital elevation model (DEM) from the 1∶25,000 topographic maps [35]. We then resampled the classification results into forest maps with a minimum mapping unit (MMU) of 1 ha.

### Data Analysis

#### Changes in forest area

Using buffer analysis in areas around the NRs, we generated layers of adjacent unprotected areas (a 10 km buffer area around the NRs’ boundaries) and the wider unprotected landscape (>10 km from NRs’ boundaries) (Fig. 1). We then measured 2000 and 2010 forest areas inside the NRs, in adjacent unprotected areas, and in the wider unprotected landscape. We chose changes in forest area in the wider unprotected landscape as controls, following [15]. To simplify the dataset and minimize statistical dependence in the dataset, we used random sampling instead of treating the whole island as a study subject. Because the sampling can only provide an estimate of the true outcome of the whole island, a sufficiently large number of sampling plots was required. Considering the minimum NR area in Hainan (100 ha), we used 100 ha quadrats as sampling plots. We randomly sampled 2000 plots of 100 ha across Hainan Island and excluded plots (*N* = 576) whose boundaries crossed the boundaries of NRs, adjacent unprotected areas, or the wider unprotected landscape. We calculated forest area for 2000 and 2010 for each plot inside NRs (*N* = 147), in adjacent unprotected areas (*N = *626), and in the wider unprotected landscape (*N = *651). We conducted a Wilcoxon signed-rank test to evaluate whether the total amount of forest area was different between 2000 and 2010 inside NRs, in adjacent unprotected areas, and in the wider unprotected landscape, respectively. We performed three Mann-Whitney *U* tests before considering the covariates to compare changes in forest areas between (1) NRs and adjacent unprotected areas; (2) NRs and wider unprotected landscape; (3) adjacent unprotected areas and wider unprotected landscape.

#### Forest fragmentation analysis

For each NR and its 10-km adjacent unprotected area, we calculated the values of fragmentation indices between 2000 and 2010. The indices we used included the mean patch size (MPS, the average forest patch size, in hectares) and the mean nearest neighbor (MNN, the average edge-edge distance between each forest patch and the nearest neighboring patch, in meters). We conducted a Wilcoxon signed-rank test to detect differences between the two periods of time in forest fragmentation index.

#### Testing the effect of covariates on forest change

For each sampling plot, we selected forest area in 2000, elevation, slope, and distance from the edge to the nearest main roads as covariates for statistical analyses, following [1], [15], [23]. We obtained data on elevation and slope from the digital elevation model (DEM) created from 1∶25,000 topographic maps. We also created digital layers of main roads (including highways, national roads, provincial roads and county roads) from 1∶25,000 topographic maps of 1997. Covariates that affect a response variable may be correlated with each other, so we used partial correlation analysis to measure the degree of association between one covariate and the response variable (change in forest area), controlling for the effects of other variables [36]. We calculated the partial correlation coefficients (*r_p_*) with the following four analyses: (1) between forest area in 2000 and changes in forest area (control variables: inside or outside of NRs, elevation, slope, and distance to nearest roads); (2) between elevation and changes in forest area (control variables: inside or outside of NRs, forest area in 2000, slope, and distance to nearest roads); (3) between slope and changes in forest area (control variables: inside or outside of NRs, forest area in 2000, elevation, and distance to nearest roads); (4) between distance to nearest roads and changes in forest area (control variables: inside or outside of NRs, forest area in 2000, elevation, and slope).

#### Testing the effectiveness of nature reserve system

We used the Pearson’s *r* correlation analysis to evaluate relationships among covariates. We then used principal component analysis (PCA) to convert these potentially correlated variables into a set of values of linearly uncorrelated variables. We selected only the first few principal components that could explain most (>80%) of the observed variance among forest area in 2000, elevation, slope, and distances to main roads, and then reduced the dimensionality of the transformed data. We then used a general linear model, the analysis of covariance, to compare changes in forest area on the condition that the effects of these new covariates be balanced between (1) NRs and adjacent unprotected areas; (2) NRs and the wider unprotected landscape; (3) adjacent unprotected areas and the wider unprotected landscape.

## Results

We produced the final forest maps of Hainan for 2000 and 2010, and they showed overall accuracy of 93.2% and 88.5%, respectively. In 2000, about 18.0% (612,830 ha) of the island was covered by tropical forests. From 2000 to 2010, the overall size of Hainan’s forests was reduced by 6.8% (41,399 ha), whereas the forest area inside the NRs increased (Wilcoxon test: *N* = 147, *W* = 55.65, *P*<0.001). In contrast, adjacent 10-km unprotected areas and the wider unprotected landscapes both experienced deforestation (*N = *626, *W* = 292.68, *P*<0.001; and *N = *651, *W* = 303.74, *P*<0.001) (Table 1, 2).

**Table 1:** Changes in tropical forests across Hainan Island.

	Area of forests (ha)		
	2000	2010	% change
Inside nature reserves	169,169	180,206	+6.5%
In adjacent unprotected areas	268,403	250,769	–6.6%
In wider unprotected landscape	175,258	140,456	–19.9%
**Total**	**612,830**	**571,431**	**–6.8%**

Changes in the area of tropical forests inside nature reserves, in adjacent unprotected areas (within 10 km of nature reserves’ boundaries), and in the wider unprotected landscapes (>10 km from nature reserves’ boundaries) in Hainan, China, from 2000 to 2010.

**Table 2:** Mean forest area.

		2000		2010		
Group	*N*	Mean	Std. Deviation	Mean	Std. Deviation	*P*–value^a^
1	147	73.41	28.12	78.18	27.46	0.000
2	626	30.54	28.24	28.53	31.80	0.000
3	651	22.80	28.39	17.76	28.67	0.000

Comparison of mean forest area (ha) between 2000 and 2010 across different sampling plots of 100 ha on Hainan Island (Group 1: inside nature reserves, Group 2: in adjacent 10-km unprotected areas, Group 3: in the wider unprotected landscape).

a Wilcoxon Signed Ranks Test (2-tailed).

The results also indicated that the forest patches inside most NRs were becoming isolated. Of the 28 NRs studied, 12 experienced decreases in MPS and 18 experienced increases in MNN. Although non-parametric testing showed that the MPS of forest patches inside the NRs underwent no changes between 2000 and 2010 (from 128.3 ha to 178.3 ha, Wilcoxon test: *N = *28, *W* = 9.80, *P* = 0.224), the MNN showed an increase (from 246.3 m to 319.8 m, *N = *28, *W* = 10.75, *P* = 0.007). The MPS and MNN of forest patches in adjacent 10-km unprotected areas both showed significant changes, from 11.2 ha to 19.7 ha (*N = *28, *W* = 6.25, *P*<0.001) and from 331.7 m to 360.2 m (*N = *28, *W* = 13.77, *P* = 0.006), respectively.

As for forest area in 2000, elevation, slope, and distance to nearest roads, correlation tests showed that all four covariates had strong or moderately-strong positive relationships with one another (*r* >0.4). The strongest correlations were between forest area in 2000 and slope (*r* = 0.789), followed by forest area in 2000 and elevation (*r* = 0.785). Elevation and slope were also strongly and positively correlated (*r* = 0.759) (*P*<0.001 in all cases). By defining control variables, the partial correlation analyses further and better explained the coefficients (*r_p_*) between the response variable (change in forest area from 2000 to 2010) and the covariates (forest area in 2000, elevation, slope, and distance to nearest roads, respectively). The variables that were most strongly correlated with the change in forest area were: forest area in 2000 (*r_p_* = –0.552) and elevation (*r_p_* = 0.442), followed by slope (*r_p_* = 0.235). Distance to nearest roads had a weaker but still significant effect (*r_p_* = 0.116) (*P*<0.001 in all cases).

Overall, the results suggested that, in terms of preventing deforestation, the NR system offered an effective solution over the past 10 years. Without considering the covariates (the simple non-parametric tests), forest area inside NRs showed an increase relative to those in adjacent 10-km unprotected areas (Mann-Whitney *U* test: *U* = 31511.00, *P*<0.001) and with those in the wider unprotected landscape (*U* = 24474.00, *P*<0.001). Furthermore, adjacent unprotected areas showed lower levels of deforestation than the wider unprotected landscape (*U* = 183996.50, *P* = 0.003). By defining control variables, the first two PCA variables, which had explained most of the variance observed (88.9%) among the four covariates, were selected as new covariates. The analysis of the general linear model still showed that deforestation level was lower inside NRs than those in adjacent unprotected areas and in the wider unprotected landscape (*P*<0.05 in all cases). However, the mean differences in the pairwise comparisons were all lower than those in the simple non-parametric tests (Table 3).

**Table 3:** Pairwise comparisons.

	Without covariates		After the covariates were balanced	
Group (I) vs. Group(J)	Mean difference (I–J)	*P*–value^a^	Mean difference (I–J)	*P*–value^b^
Group 1 vs. Group 2	6.78**	0.000	4.52*	0.006
Group 1 vs. Group 3	9.81**	0.000	7.17**	0.000
Group 2 vs. Group 3	3.03*	0.003	2.65	0.004

The results of pairwise comparisons before and after the effects of covariates were balanced. The dependent variable was the changes in forest area (ha). Group identity served as the independent variable (Group 1: inside nature reserves, Group 2: in adjacent 10-km unprotected areas, Group 3: in the wider unprotected landscape). The covariates were the first and second components extracted from the principal component analysis of the independent variables (forest area in 2000, elevation, slope, and distance to nearest roads).

* The mean difference is significant at the 0.05 level.

** The mean difference is significant at the 0.001 level.

a Non–parametric tests (Mann–Whitney U tests).

b Adjustment for multiple comparisons: Least Significant Difference.

## Discussion

Evaluating the effectiveness of NRs for the purpose of conserving tropical forests is urgent. Previous reports have analyzed trends at the level of the NR system (rather than at the individual NR level), using analysis to balance the effects of the covariates that might affect changes in forest area and determining whether deforestation activities had been displaced from NRs onto adjacent unprotected areas (rather than a simple inside-outside comparison) [5]–[7], [12]–[17]. We addressed these points in the case of the tropical Hainan Island by dividing the natural forests into three groups (NRs, adjacent 10-km unprotected areas, and the wider unprotected landscapes) and comparing the differences in changes in forest area among these groups before and after the effects of given covariates (historical area and accessibility of natural forests) were balanced.

First, there were increases in forest area inside Hainan’s NR system, implying that forest recovery could be relatively fast and efficient in the tropics even over a relatively short, 10-year time frame [37]–[39]. Preconditions of the recovery should include the absence of human disturbance, and proximity of sufficient amount of native trees needed for regeneration [28], [31]. The ban on the clear-cutting of all natural forests since 1994 may also have favored the recovery [31]. In contrast, the unprotected areas (including the adjacent unprotected areas and the wider unprotected landscapes) showed an 11.8% loss (1.18% year^–1^) in forest over, which was not as severe as that observed in other tropical regions [5]. As in the rest of the tropical world, the main reasons were shifting cultivation and illegal logging outside of NRs [40], [41]. The tendency was robust regardless of whether the effects of historical forest area and accessibility of natural forests were taken into account or not. Given that the changes in forest area had strong correlation with forest area in 2000 (negative) and elevation (positive), controlling the effects of these covariates is important to better explain the effectiveness of NR system for conserving tropical forests. This result concurred with those of studies in Costa Rica [1] and in Sumatra [15]: the simple inside-outside comparisons may have considerably overestimated the protective effects of NRs, particularly where NRs showed marked topographic differences from their immediate surroundings [42].

The results also suggested the absence of a detrimental “neighborhood leakage” effect on Hainan Island. Even though the adjacent unprotected areas experienced some deforestation, they saw less amounts of deforestation than the wider unprotected landscapes. Population growth, pre-emptive clearing, and the relocation of illegal settlers along the boundaries of the NRs may have a marginal influence on deforestation, as in Sumatra [15]. This could be explained by the fact that most of Hainan’s NRs and their adjacent unprotected areas are located in the central mountainous region, which has high elevations and steep slopes, and these areas are less subject to human activity [33].

The recovered forest within NRs’ boundaries cannot be assumed to have reached the full naturalness of the former mature rainforest [43]. In fact, the results revealed increasing isolation of forest patches among most Hainan’s NRs (>60%). The reasons could be the selective logging at higher elevations and the conversion of smaller forest patches to commercial plantations (e.g. rubber and eucalyptus) in the lowlands [29]. In addition, short term regeneration can only fulfill some of the functions of mature rainforests [44]–[46]. Carbon sequestration might continue with little changes after regeneration [45], whereas supportive functions, such as providing wildlife habitat, may be altered [38], [44]. For example, even though some monoculture pine plantations (classified as non-forest in this study) in the Bawangling National Nature Reserve have been restored to mixed native forests, the Hainan gibbon (*Nomascus hainanus*), one of the world’s rarest apes, still lost some of its prime habitat [29], [39].

In sum, these results suggested that Hainan’s NR system was effective in preventing deforestation over the past 10 years, but attention should be paid to future conservation efforts because other factors (the covariates in our study) were also correlated with deforestation. We assume that sites with less challenging environmental conditions (e.g. low elevation, flatter slopes) or proximity to roads will have the potential for rapid deforestation because of their attractiveness to logging operations, and that this would be especially true for remaining mature rainforest at lower elevations. Establishing NRs with effective management in the lowlands and preventing illegal selective logging within existing NRs will provide powerful instruments to prevent deforestation. Moreover, large-scale restoration of native forests is required to insure that the regenerated forests within the NR system regain functionality. This could help connect fragmented patches of forest, an issue of special importance to many local threatened and endangered species, such as the Hainan Gibbon [33], [39].

## Supporting Information

Table S1 Nature reserves dedicated primarily to the protection of forest ecosystems on Hainan Island.
(DOC)
