# Supplementary material for: Effectiveness of Nature Reserve System for Conserving Tropical Forests: A Statistical Evaluation of Hainan Island, China
Source: PLoS One. 2013 Feb 28;8(2):e57561. doi: 10.1371/journal.pone.0057561 (PMC3585332; doi:10.1371/journal.pone.0057561)
Supplement: Table S1 — Nature reserves dedicated primarily to the protection of forest ecosystems on Hainan Island. (DOC) [file pone.0057561.s001.doc]

**Table S1:**

**Nature reserves mainly dedicated to protect forest ecosystem of Hainan Island.**

| **Name of nature reserves** | **Location**  **(county)** | **Area**  **(ha)** | **Conservation target** | **Year of establishment** |
| --- | --- | --- | --- | --- |
| Fuwan | Sanya | 3,325 | Forest ecosystem | 1974 |
| Jianfengling | Ledong | 20,170 | Tropical seasonal rainforest | 1976 |
| Bangxi | Baisha | 358 | Wild animals and habitats | 1976 |
| Nanwan | Lingshui | 1,007 | Wild animals and habitats | 1976 |
| Datian | Dongfang | 1,314 | Wild animals and habitats | 1976 |
| Bawangling | Changjiang | 29,980 | Forest ecosystem | 1980 |
| Liji | Wanning | 925 | Tropical seasonal rainforest | 1980 |
| Huishan | Qionghai | 4,325 | Tropical seasonal rainforest | 1981 |
| Jiaxin | Wanning | 7,588 | Tropical seasonal rainforest | 1981 |
| Shangxi | Wanning | 11,662 | Tropical seasonal rainforest | 1981 |
| Fanjia | Danzhou | 4,660 | Tropical seasonal rainforest | 1981 |
| Jianling | Wanning | 10,923 | Tropical seasonal rainforest | 1981 |
| Liulianling | Wanning | 2,746 | Tropical seasonal rainforest | 1981 |
| Nanlin | Wanning | 5,775 | Tropical seasonal rainforest | 1981 |
| Jiaxi | Ledong | 8,327 | Tropical seasonal rainforest | 1981 |
| Baishiling | Qionghai | 747 | Forest ecosystem | 1982 |
| Dahuajiao | Wanning | 106 | Wild animals and habitats | 1984 |
| Diaoluoshan | Lingshui | 18,389 | Tropical rainforest | 1984 |
| Wuzhishan | Qiongzhong | 13,436 | Tropical natural forest | 1985 |
| Ganshiling | Sanya | 1,715 | Wild plants | 1985 |
| Tunchang | Tunchang | 100 | Wild animals and habitats | 1986 |
| Baoguoshan | Ledong | 181 | Forest ecosystem | 1992 |
| Liudao | Sanya | 1,800 | Tropical seasonal rainforest | 1996 |
| Mihouling | Dongfang | 12,215 | Tropical rainforest | 2004 |
| Yinggeling | Qiongzhong | 50,630 | Tropical seasonal rainforest | 2004 |
| Limushan | Qiongzhong | 12,889 | Tropical seasonal rainforest | 2004 |
| Dongfang | Dongfang | 1,429 | Wild animals and habitats | 2006 |
| Baomeiling | Changjiang | 3,845 | Tropical rainforest | 2006 |
